# Gene Expression-Based Inference of Metabolic Signatures Reveals Distinct Molecular Profiles in Right- and Left-Sided Colon Cancer

**DOI:** 10.3390/metabo15120768

**Published:** 2025-11-27

**Authors:** Ismail Ertuğrul, Ayşe Büşranur Çelik, Mervenur Al, Mustafa Duman, Yunus Emre Altuntaş, Erdal Polat, Yunus Emre Ertuğrul, Hasan Fehmi Küçük, Yusuf Tutar

**Affiliations:** 1 Department of General Surgery, Kartal Dr. Lutfi Kırdar City Hospital, 34865 Istanbul, Turkey; yunusemre.altuntas@sbu.edu.tr (Y.E.A.); hasanfehmi.kucuk@sbu.edu.tr (H.F.K.); 2 Department of Molecular Biology and Genetics, Hamidiye Institute of Health Sciences, University of Health Sciences, 34668 Istanbul, Turkey; aysebusranur_celik25@erdogan.edu.tr; 3 Department of Medicinal Biochemistry, Faculty of Medicine, Recep Tayyip Erdoğan University, 53020 Rize, Turkey; 4 Division of Medicinal Biochemistry, Department of Basic Medical Sciences, University of Health Sciences, 34668 Istanbul, Turkey; biochemistry.merve@gmail.com; 5 Department of Gastroenterology Surgery, Koşuyolu High Specialization Education and Research Hospital, 34846 Istanbul, Turkey; mustafa.duman@sbu.edu.tr (M.D.); erdal.polat@sbu.edu.tr (E.P.); 6 Medical School, Maltepe University, 34857 Istanbul, Turkey; yun.bjk.44@gmail.com; 7 Division of Medicinal Biochemistry, Department of Basic Medical Sciences, Faculty of Medicine, Recep Tayyip Erdogan University, 53020 Rize, Turkey; yusuf.tutar@erdogan.edu.tr; 8 Training and Research Hospital, Recep Tayyip Erdoğan University, 53020 Rize, Turkey; 9 Medical Oncology Program, Recep Tayyip Erdogan University, 53100 Rize, Turkey; 10 Molecular Medicine Program, Recep Tayyip Erdogan University, 53100 Rize, Turkey

**Keywords:** colon cancer, signaling pathways, molecular mechanisms

## Abstract

**Background/Objective**: Colon cancer, the third most diagnosed cancer worldwide, is anatomically classified into right- and left-sided colon cancers based on embryonic origin and vascular supply. The aim of this study was to investigate molecular differences between patients with right- and left-sided colon cancer. **Methods**: In this pilot study, Blood samples from right-sided (*n* = 6) and left-sided (*n* = 6) colon cancer patients, as well as healthy controls (*n* = 6), were analyzed for 92 cancer-related genes via RT-qPCR. KEGG pathway analysis was performed with ShinyGO 0.82, and gene–metabolite interactions were assessed using EnrichR and MetaboAnalyst 6.0. Additionally, patients’ sociodemographic and clinical data were analyzed. **Results**: KEGG analysis revealed that p53, HIF-1, TNF, PI3K/Akt, MAPK, and Rap1 signaling pathways were enriched in right-sided colon cancer, whereas VEGF, HIF-1, MAPK, PI3K/Akt, Rap1, and Ras signaling pathways were implicated in left-sided colon cancer. In the gene–metabolite analysis, key metabolites identified in right-sided colon cancer included palmitic acid, adenosine triphosphate (ATP), glycerol, and adenosine diphosphate (ADP), associated with genes such as *ACSL4*, *TP53*, *MAPK14*, *FLT1*, *AURKA*, *KDR*, *ERCC3*, and *PFKL*. For left-sided colon cancer, glucose-6-phosphate (G6P), ATP, ADP, glycerol, and palmitoyl-CoA were key metabolites forming the basis of the gene–metabolite network, along with genes including *G6PD*, *PFKL*, *MAPK14*, *FLT1*, *CDK4*, *AURKA*, *MAP2K1*, *ERCC3*, *TP53*, *WEE1*, and *GPD2*. **Conclusions**: These findings highlight distinct molecular profiles between right- and left-sided colon cancers, particularly in pathways related to angiogenesis, apoptosis, ferroptosis, and fatty acid metabolism, which may inform therapeutic strategies.

## 1. Introduction

Colorectal cancer ranks as the third most diagnosed malignancy worldwide, following lung and breast cancer [1]. While approximately 70–80% of colorectal cancers develop sporadically, 20–30% arise in the context of hereditary predisposition [2]. Colorectal cancer typically originates from adenomatous polyps, which, though initially benign, may undergo malignant transformation over time, ultimately progressing to invasive cancer through a process known as the “adenoma–carcinoma sequence” [3].

A variety of genetic and epigenetic alterations play critical roles in the molecular pathogenesis of colorectal cancer. Among the most frequently observed genetic alterations are mutations in the APC, KRAS, BRAF, p53, and mismatch repair (MMR) genes [4]. These molecular aberrations are crucial for subclassifying colorectal cancers and guiding targeted therapeutic strategies [5].

Anatomically and embryologically, the colon exhibits notable differences along its longitudinal axis. The right colon (comprising the cecum, ascending colon, and hepatic flexure) originates from the midgut and receives its vascular supply from the superior mesenteric artery. In contrast, the left colon (including the splenic flexure, descending colon, and sigmoid colon) is derived from the hindgut and is supplied by the inferior mesenteric artery [6]. These embryological and vascular distinctions have important clinical implications. From a pathological standpoint, lesions such as sessile serrated adenomas and mucinous adenocarcinomas are more frequently encountered in the right colon. In contrast, tubular adenomas and conventional adenocarcinomas predominate in the left colon [7]. These differences—embryological, anatomopathological, and microbiome-based—bear clinical relevance. For example, right-sided tumors may present later due to subtler symptoms such as occult bleeding or anemia. In contrast, left-sided tumors, owing to their growth in a narrower lumen, often manifest earlier with symptoms like altered bowel habits, obstruction, or visible rectal bleeding. Therefore, tumor localization should be carefully considered during treatment planning and prognosis assessment.

Colorectal cancer is classically characterized by a multistep process of genetic and epigenetic transformation, known as the “adenoma–carcinoma sequence” [3]. Mutations in genes such as *APC*, *KRAS*, and *TP53* are commonly involved in this progression. Beyond these mutations, two principal forms of genomic instability have been defined in colorectal carcinogenesis: chromosomal instability (CIN) and microsatellite instability (MSI). Epigenetic mechanisms, including DNA methylation, histone modifications, and chromatin remodeling, further contribute to tumor development. The tumor microenvironment also plays a pivotal role in colorectal cancer progression; stromal cells, immune infiltrates, and the extracellular matrix surrounding the tumor significantly influence its growth and metastatic potential [8].

It has long been recognized that the molecular pathways activated in colorectal tumors differ according to their location in the right or left colon. These disparities manifest in numerous biological domains, including DNA repair mechanisms, chromatin organization, transcription factor dynamics, metabolic reprogramming, exosomal content, immune evasion, angiogenesis, epigenetic regulation, alternative splicing, telomere biology, and cellular senescence. Comprehensive genomic databases have cataloged over 2000 genes associated with colorectal cancer. However, more focused analyses typically emphasize approximately 20–30 well-characterized genes, such as *APC*, *MMR*, *KRAS*, *NRAS*, *BRAF*, *TP53*, and *STK11*.

Due to the prohibitive cost of large-scale genomic profiling in individual patients, we conducted a targeted analysis of 92 genes, referred to as “junction genes,” which intersect multiple oncogenic pathways. These genes are critically involved in key cellular processes, including angiogenesis, apoptosis, cell cycle regulation, DNA repair, and metabolism. Alterations in these genes may contribute to tumor growth, metastasis, and therapeutic resistance. A deeper understanding of these genes holds potential for improving colorectal cancer diagnosis, prognosis, and treatment. Moreover, their analysis may facilitate the development of personalized therapeutic strategies.

This study hypothesizes that right- and left-sided colon cancers exhibit distinct gene expression and metabolite profiles that may be associated with differences in pathway activation and potential therapeutic response.

## 2. Materials and Methods

### 2.1. Patients and Blood Samples

Ethical approval for the study was obtained from the Ethics Committee of the University of Health Sciences, Istanbul Kartal Dr. Lütfi Kırdar City Hospital (Ethics Committee No: 19.07.2023/514/254/5). Between May and June 2024, the sample was composed of patients diagnosed with right-colon (*n* = 6) and left-colon (*n* = 6) tumors, along with healthy individuals (*n* = 6) with normal colonoscopy results. All patients were over 18 years of age, and those with metastasis, receiving neoadjuvant therapy, or presenting as emergency cases were excluded from the study. In addition to sociodemographic data such as age, gender, and BMI, clinical data were also collected. Blood samples obtained from patients were transported under appropriate cold-chain conditions, and isolation procedures were performed. The commercial kit only isolates RNA from peripheral blood samples but does not isolate other RNAs, including circulating RNAs; therefore, the RNAs analyzed represent only cell-derived transcripts rather than circulating cell-free RNAs.

### 2.2. Real-Time Quantitative Polymerase Chain Reaction (RT-qPCR) and Gene Enrichment Analysis

RNA isolation from the collected blood samples was performed using the innuPREP Blood RNA Kit 2.0 (Innuscreen GmbH, Berlin, Germany), and cDNA synthesis was carried out using the Wonder RT cDNA Synthesis Kit (Euroclone, Milan, Italy) according to the manufacturer’s instructions. To investigate the molecular differences between right and left colon cancer patients, expression changes of 92 cancer-related genes (Supplementary Table S1) were determined by RT-qPCR (Analytik Jena QTower3, Jena, Germany) using the FluoCycle II™ SYBR^®^ Master Mix kit. *GAPDH* and *ACTINB* were used as reference genes, and the results were analyzed using the 2^−ΔΔCT^ method [9]. Genes showing significant expression changes (≥2-fold upregulation or ≤0.5-fold downregulation) in both groups were identified and subjected to gene enrichment analysis using the KEGG database in ShinyGO v0.82 [10]. The results were ranked by false discovery rates (FDRs).

### 2.3. Gene-Metabolite Interaction

The metabolites associated with the genes that showed significant differential expression in the RT-qPCR analysis were analyzed using the EnrichR platform with the Metabolomics Workbench Metabolites 2022 database, and the relevant metabolites were identified based on *p*-values [11]. The relationship between the identified metabolites and genes was evaluated using the MetaboAnalyst 6.0 database [12].

### 2.4. Statistics

All statistical analyses were performed using GraphPad Prism software version 8.0.2 (GraphPad Software, San Diego, CA, USA). The control group was set to a relative expression value of 1, and fold change values were calculated using the 2^−ΔΔCt^ method. Data were expressed as mean ± standard deviation (SD), and *p* < 0.05 was considered statistically significant (*p* < 0.05; *p* < 0.01, *; *p* < 0.001, ***).

## 3. Results

### 3.1. Analysis of Sociodemographic and Clinical Parameters

The sociodemographic and clinical characteristics of the total of 18 patients included in the study are presented in Table 1. The overall mean age of the patients was 58.77 ± 11.17 years, with an age range between 36 and 72 years. The majority of patients were male (72.2%), and the average height and weight were 166.72 ± 9.56 cm and 75.72 ± 13.25 kg, respectively. The mean BMI was calculated as 27.20 ± 3.70. Regarding tumor localization, the most frequent sites were the sigmoid colon (25%) and the right colon (25%). Smoking and alcohol use were both reported at rates of 11.1%. Among surgical procedures, the most common were LAR (27.8%) and right hemicolectomy (27.8%). Additionally, 33.3% of the patients did not undergo any surgery.

In the left colon group, the mean age was 63.83 ± 10.57 years, with an age range of 44 to 76 years. The majority of patients were male (83.3%), and the average height and weight were 172.00 ± 4.85 cm and 81.83 ± 9.28 kg, respectively. The mean BMI was calculated as 27.72 ± 2.93. Regarding tumor localization, the most frequent sites were the sigmoid colon (50%) and the rectosigmoid colon (33.3%). Smoking and alcohol use were both reported at rates of 16.7%. Among the surgical procedures, lar (83.3%) and anterior resection (16.7%) were the most performed.

In the right colon group, the mean age was 63.16 ± 8.01 years, with an age range between 55 and 73 years. The majority of patients were male (66.7%), and the average height and weight were 164.00 ± 12.63 cm and 76.33 ± 17.94 kg, respectively. The mean BMI was calculated as 28.25 ± 4.64. The most common tumor localizations were the right colon (50%) and the ileocecal valve (33.3%). Smoking and alcohol use were both reported at rates of 16.7%. Regarding surgical procedures, the right hemicolectomy (83.3%) and extended right hemicolectomy (16.7%) were the most commonly performed.

In the normal patient group, the mean age was 49.33 ± 9.37 years, with an age range of 36 to 63 years. The majority of patients were male (66.7%), and the average height and weight were 164.16 ± 8.81 cm and 69.00 ± 8.46 kg, respectively. The mean BMI was calculated as 25.62 ± 3.44. No smoking or alcohol use was detected. No surgical procedures were performed.

### 3.2. Gene Expression Analysis in Right and Left Colon Cancer

**Figure 1:**
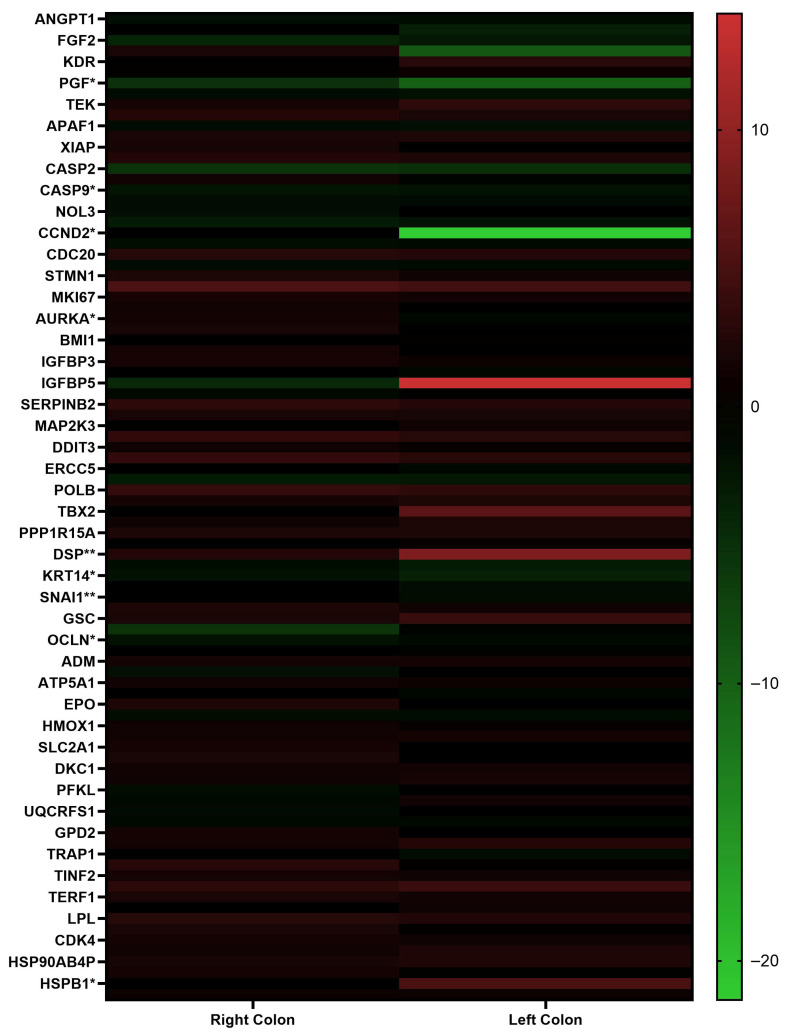
Heatmap representation of differentially expressed genes in right and left colon cancer patients based on RT-qPCR results. The analysis was performed and visualized using GraphPad Prism 8.0.2 software. Red indicates upregulated genes, while green indicates downregulated genes. Statistical significance between right and left colon cancer groups was determined using the Mann–Whitney U test (* *p* < 0.05, ** *p* < 0.01).

Total RNA was isolated from blood samples obtained from patients with right- and left-sided colon cancer. Expression changes in genes involved in key cellular processes such as apoptosis, angiogenesis, cell cycle regulation, and metabolism (Supplementary Table S1) were analyzed by RT-qPCR (Figure 1, Supplementary Tables S2 and S3).

The results obtained from patients with right and left colon cancer were analyzed using the Mann–Whitney U test. A statistically significant difference was observed between the two groups in the expression levels of the following genes: *ANGPT2*, *FLT1*, *PGF*, *CASP9*, *CCND2*, *MCM2*, *AURKA*, *DDB2*, *ERCC3*, *GADD45G*, *DSP*, *KRT14*, *SNAI1*, *OCLN*, *HSPB1*, and *CA9.*

### 3.3. Gene Enrichment Analysis

**Figure 2:**
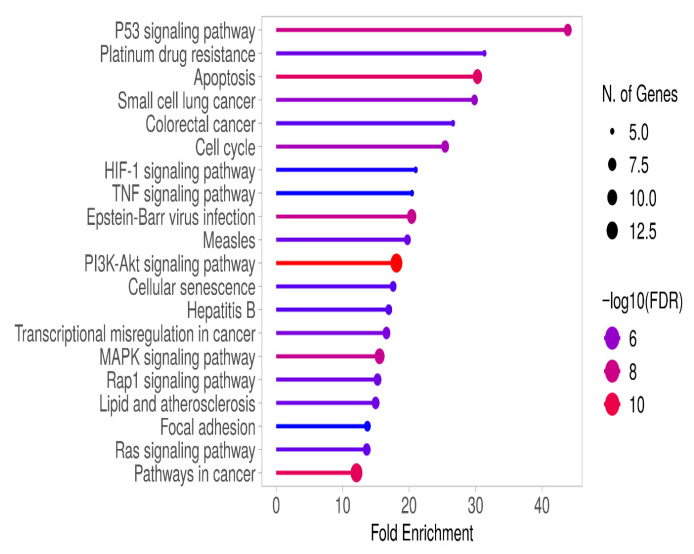
Gene enrichment analysis of RT-qPCR results from right colon cancer patients was performed using KEGG analysis on the ShineyGO 0.82 platform. The associated pathways were selected based on FDR values and ranked by fold enrichment.

Genes with significantly altered expression (≥2-fold increase or ≤0.5-fold decrease) between right and left colon cancer patients, as determined by RT-qPCR analysis, were subjected to KEGG pathway analysis using the ShinyGO 0.82 platform. In right colon cancer patients, cellular processes such as apoptosis, cell cycle, and senescence were associated with signaling pathways including p53, HIF-1, TNF, PI3K/AKT, MAPK, and RAP1 (Figure 2 and Supplementary Table S4).

**Figure 3:**
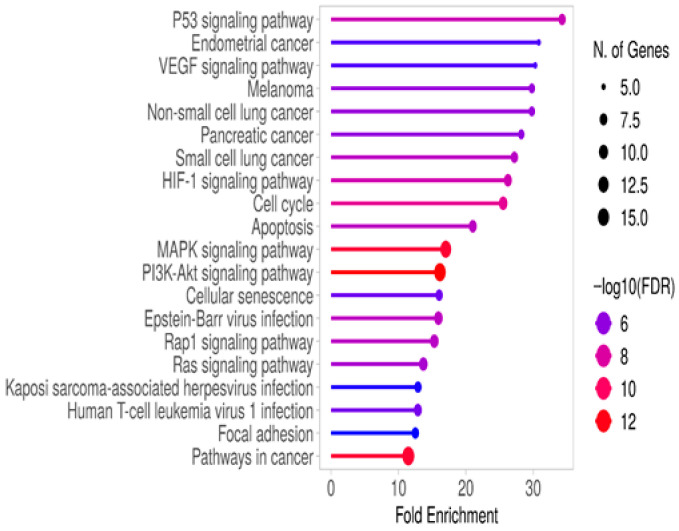
Gene enrichment analysis of RT-qPCR results from left colon cancer patients was performed using KEGG analysis on the ShinyGO 0.82 platform. The associated pathways were selected based on FDR values and ranked by fold enrichment.

In left colon cancer patients, KEGG analysis revealed similar pathways as in right colon cancer but with a different ranking order. The pathways involved included p53, VEGF, HIF-1, MAPK, PI3K/Akt, Rap1, and Ras signaling, as well as cell cycle, apoptosis, and senescence processes (Figure 3 and Supplementary Table S5).

### 3.4. Gene-Metabolite Interactions

Metabolites associated with significantly differentially expressed genes identified by RT-qPCR analysis were determined using the EnrichR Metabolomics Workbench Metabolite 2022 database (Supplementary Tables S6 and S7). The relationships between the identified metabolites and genes were further analyzed using the MetaboAnalyst 6.0 database.

**Figure 4:**
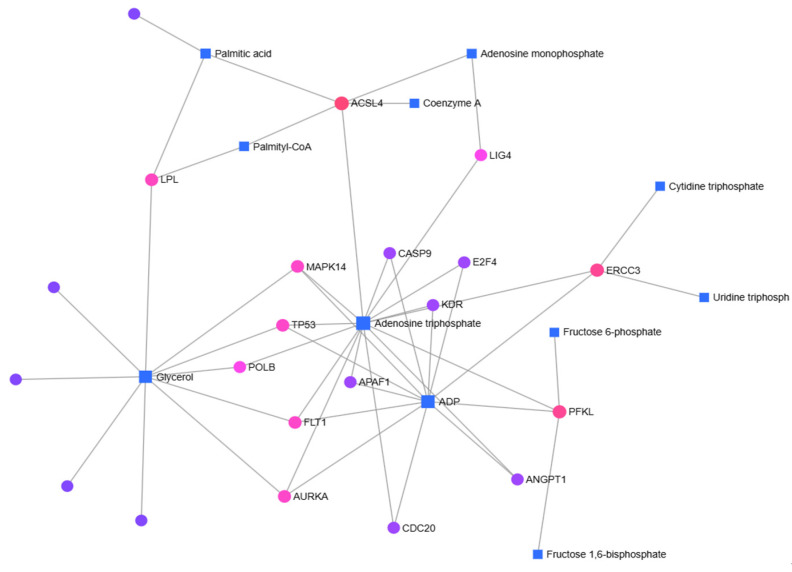
In right-sided colon cancer patients, gene–metabolite interactions were identified using the MetaboAnalyst 6.0 database.

In right-sided colon cancer, Palmitic acid, Adenosine triphosphate (ATP), Glycerol, and Adenosine diphosphate (ADP) were identified as key metabolites, with *ACSL4*, *TP53*, *MAPK14*, *FLT1*, *AURKA*, *KDR*, *ERCC3*, and *PFKL* genes forming the basis of gene–metabolite interactions (Figure 4).

In left-sided colon cancer, Glucose-6-phosphate (G6P), ATP, ADP, Glycerol, and Palmitoyl CoA were identified as key metabolites forming the basis of the gene–metabolite network, along with the genes *G6PD*, *PFKL*, *MAPK14*, *FLT1*, *CDK4*, *AURKA*, *MAP2K1*, *ERCC3*, *TP53*, *WEE1*, and *GPD2*.

## 4. Discussion

Colorectal cancer is the third most diagnosed malignancy worldwide [1]. In recent years, it has been demonstrated that colorectal tumors exhibit significant clinical, histological, and molecular differences depending on their localization in the right or left colon. The right colon, comprising the cecum, ascending colon, and hepatic flexure, and the left colon, including the splenic flexure, descending colon, and sigmoid colon, are supplied by different arteries, which contribute to the distinct biological behavior observed in tumors arising from these regions [6]. Moreover, right-sided colon tumors are often diagnosed at a later stage due to subtle symptoms such as occult bleeding or anemia, whereas left-sided tumors, growing within a narrower lumen, tend to present earlier with more evident clinical signs, such as altered bowel habits, obstruction, or visible rectal bleeding. Therefore, tumor localization should be carefully considered in treatment planning and prognostic evaluation.

Molecular analysis results between right- and left-sided colon cancers generally revealed similar patterns [13,14]. Among the genes that were found to be statistically significant based on RT-qPCR analysis, Angiopoietin 2 (*ANGPT2*), Fms-Related Receptor Tyrosine Kinase 1 (*FLT1*, *VEGFR*), and Placental Growth Factor (*PGF*) are associated with angiogenesis. The expression of all three genes was found to be more profoundly affected and significantly downregulated in left-sided colon cancer (Supplementary Tables S2 and S3). Notably, the KEGG analysis identified the Vascular Endothelial Growth Factor (VEGF) signaling pathway only in left-sided colon cancer (Figure 3). Previous studies have shown that tumor progression in metastatic colon cancer is associated with VEGF and EGFR (Epidermal Growth Factor Receptor) signaling, and that inhibitors targeting the VEGF family are used in colon cancer treatment [15]. FLT1, a member of the VEGF receptor (VEGFR) family, along with ANGPT2 and PGF, plays a role in vascular development and contributes to colon cancer progression [16,17]. Jary et al. reported that *ANGPT2* expression increases with advanced stages of colon cancer, although its expression generally remains low in both right- and left-sided colon cancers, and that higher expression levels are associated with poor prognosis. In our study, we similarly observed significantly reduced expression of *ANGPT2* in both right- and left-sided colon cancers, with *FLT1* and *PGF* showing even lower expression in left-sided colon cancer.

Angiogenesis is regulated by both the *VEGF* and *PGF* families and is further promoted by hypoxia-inducible factor 1-alpha (HIF-1α) activation [15]. In line with this, HIF-1 signaling was identified in our gene enrichment analyses (Figure 2 and Figure 3). In addition to *ANGPT2*, *FLT1*, and *PGF*, we also found that *CA9*, a known HIF-1 target gene, did not show a significant difference in right-sided colon cancer but was strongly downregulated in left-sided colon cancer, with a 23.15-fold decrease in expression. In previous studies on colorectal cancer, high expression of *CA9* has been associated with poor outcomes in cancer patients [18,19]. In our study, the lower expression of *CA9* in left colon cancer suggests that it may respond better to treatment in this regard.

The *HSPB1* (Heat Shock Protein Beta-1) gene, which showed a significant change in right and left colon cancer, is known as a negative regulator of ferroptosis, one of the programmed cell deaths, and its high expression has been shown to influence drug resistance and cancer progression [5,6]. In our study, a 20-fold increase in left colon cancer was observed, suggesting that left colon cancer may be more prone to developing drug resistance or escaping ferroptosis. Caspase 9, which plays a role in apoptosis—a type of programmed cell death—initiates the intrinsic pathway. In cancer cells, it is generally downregulated, thereby preventing cells from undergoing apoptosis [20,21]. In our study, the approximately 3-fold decrease observed specifically in left-sided colon cancer suggests that *CASP9* may be a potential suppressor in left-sided colon cancer.

**Figure 5:**
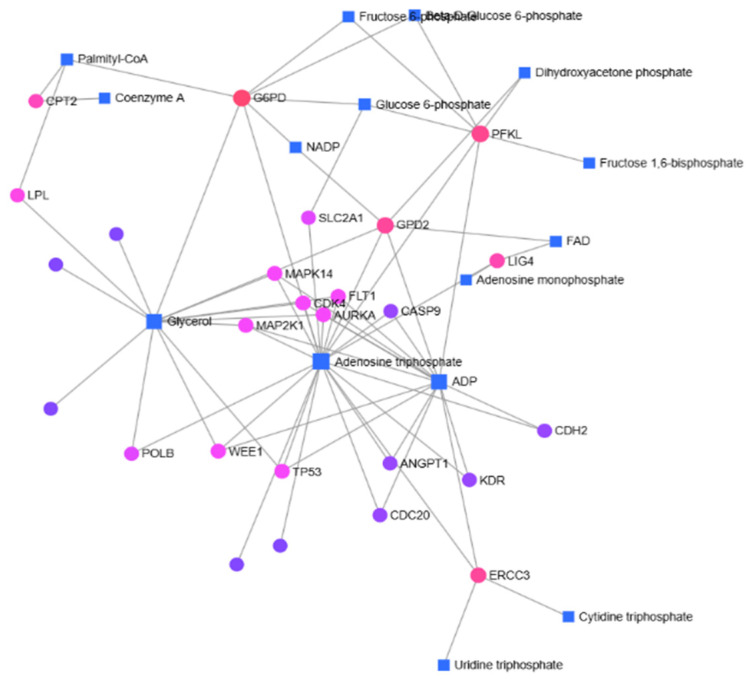
In patients with left-sided colon cancer, gene–metabolite interactions were identified using the MetaboAnalyst 6.0 database.

In the gene-metabolite interaction map, genes and metabolites associated with fatty acid metabolism were identified (Figure 4 and Figure 5). The carnitine palmitoyltransferase (CPT) family, which is commonly identified in both right and left colon, is located in the inner mitochondrial membrane and functions in fatty acid oxidation [22]. There are contradictory results in the literature regarding colorectal cancer patients [22,23,24]. In addition to the gene-metabolite map, we determined that the expression of *CPT2* was significantly increased (6.56) in left colon cancer based on RT-qPCR results. Palmitic acid, an important fatty acid in fatty acid metabolism, is a saturated fatty acid that constitutes 20–30% of the body’s fatty acids [25]. Palmitic acid, which is the first to form as a result of fatty acid synthesis, is later activated by CoA and converted to palmitoyl-CoA [26]. While palmitic acid was identified in right colon cancer, palmitoyl-CoA was identified in left colon cancer. It can be considered that palmitic acid identified in right colon cancer is more involved in lipid synthesis, whereas in left colon cancer, where palmitoyl-CoA was detected, energy production is more prioritized.

In conclusion, this study demonstrates significant molecular heterogeneity between right- and left-sided colon cancers, particularly in key oncogenic pathways such as angiogenesis, apoptosis, ferroptosis, and fatty acid metabolism. These differences underline the importance of considering tumor localization in clinical management and therapeutic decision-making for colorectal cancer patients. Further investigations into the distinct molecular mechanisms governing tumor behavior on each side of the colon may pave the way for more effective, personalized treatment strategies and improved patient outcomes.

This study has several limitations that should be acknowledged. First and foremost, the sample size was relatively small (n = 6 per group), which limits the statistical power and generalizability of the findings. As a result, the observed gene expression and pathway differences should be interpreted as preliminary. Second, RNA was isolated from peripheral blood samples rather than directly from tumor tissues, which may not fully capture tumor-specific molecular alterations, particularly for genes with limited expression in circulating blood cells. Thus, the detected expression changes may represent systemic host responses to tumor presence, including immune modulation or metabolic alterations, rather than direct tumor-derived transcripts. Third, although differential gene expression was identified, no additional validation at the protein level (e.g., Western blot, immunohistochemistry) or through independent datasets (e.g., TCGA) was performed, limiting the biological robustness of the results. Furthermore, the study design was cross-sectional, precluding any longitudinal assessment of molecular dynamics over time or in response to treatment. Lastly, while multiple statistical comparisons were conducted, details on the correction method for multiple hypothesis testing (e.g., FDR adjustment) were not fully described, which may increase the risk of false positives. Future studies with larger, well-annotated cohorts and tumor-derived samples are needed to validate and expand upon these initial findings. Finally, this study did not include direct metabolite profiling by mass spectrometry, which limits the ability to directly link gene expression changes to metabolite alterations

## 5. Conclusions

This study revealed distinct molecular signatures between right- and left-sided colon cancers, highlighting differences in key oncogenic pathways such as angiogenesis, apoptosis, ferroptosis, and fatty acid metabolism. Right-sided tumors were characterized by altered expression of genes and metabolites linked to lipid synthesis and apoptotic regulation, whereas left-sided tumors showed prominent changes in VEGF signaling, hypoxia response, and fatty acid oxidation. These findings underscore the biological heterogeneity of colon cancer based on tumor localization and support the consideration of anatomical site in therapeutic decision-making. While the results provide preliminary insights into molecular distinctions that may influence prognosis and treatment response, validation in larger, tissue-based cohorts with protein-level and longitudinal analyses is warranted. Such efforts may ultimately contribute to personalized strategies for the management of colorectal cancer.

## Figures and Tables

**Table 1:** Sociodemographic and clinical parameters.

Parameters	Total (*n* = 18)	Left Colon (*n* = 6)	Right Colon (*n* = 6)	Healthy (*n* = 6)
Age				
Mean ± SD	58.77 ± 11.17	63.83 ± 10.57	63.16 ± 8.01	49.33 ± 9.37
Median (min-max)	60.0 (36–72)	65.5 (44–76)	63.0 (55–73)	51.0 (36–63)
Sex				
Female	13 (72.2%)	5 (83.3%)	4 (66.7%)	4 (66.7%)
Male	5 (27.8%)	1 (16.7%)	2 (33.3%)	2 (33.3%)
Height				
Mean ± SD	166.72 ± 9.56	172.0 ± 4.85	164.00 ± 12.63	164.16 ± 8.81
Median (min-max)	167.0 (149–185)	173.0 (163–177)	165.0 (149–185)	163.5 (155–175)
Weight				
Mean ± SD	75.72 ± 13.25	81.83 ± 9.28	76.33 ± 17.94	69.00 ± 9.46
Median (min-max)	75.0 (55–110)	82.0 (70–95)	70.5 (62–110)	72.0 (55–78)
BMI				
Mean ± SD	27.2 ± 3.7	27.72 ± 2.93	28.25 ± 4.64	25.62 ± 3.44
Median (min-max)	27.2 (22.1–34.1)	27.5 (22.8–31.7)	28.6 (22.1–34.1)	24.4 (22.8–32.1)
Diagnosis				
Cecum tumor	1 (5.6%)		1 (16.7%)	
Ascending colon tumor	3 (16.7%)		3 (50.0%)	
Hepatic flux tumor	1 (5.6%)		1 (16.7%)	
Normal	6 (33.3%)			6 (100.0%)
Rectosigmoid tumor	1 (5.6%)	1 (16.7%)		
Rectum tumor	1 (5.6%)	1 (16.7%)		
Right colon tumor	1 (5.6%)		1 (16.7%)	
Sigmoid colon tumor	4 (22.2%)	4 (66.7%)		
Tumor location				
Cecum	1 (8.3%)		1 (16.7%)	
Ileocecal valve	2 (16.6%)		2 (33.3%)	
Rectosigmoid colon	2 (16.6%)	2 (33.3%)		
Right colon	3 (25.0%)		3 (50.0%)	
Sigmoid colon	3 (25.0%)	3 (50.0%)		
Left colon	1 (8.3%)	1 (16.7%)		
Smoking				
No	16 (88.9%)	5 (83.3%)	5 (83.3%)	6 (100.0%)
Yes	2 (11.1%)	1 (16.7%)	1 (16.7%)	0 (0.0%)
Alcohol consumption				
No	16 (88.9%)	5 (83.3%)	5 (83.3%)	6 (100.0%)
Minimal	2 (11.1%)	1 (16.7%)	1 (16.7%)	0 (0.0%)
Surgery performed				
Anterior resection	1 (5.6%)	1 (16.7%)		
Extended right hemicolectomy	1 (5.6%)		1 (16.7%)	
Low anterior resection (LAR)	5 (27.8%)	5 (83.3%)		
Right hemicolectomy	5 (27.8%)		5 (83.3%)	
None	6 (33.3%)			6 (100.0%)

## Data Availability

All data generated or analyzed during this study are included in this published article and Supplementary Materials.

## Supplementary Materials

The following supporting information can be downloaded at: https://www.mdpi.com/article/10.3390/metabo15120768/s1. Table S1: List of primers grouped according to their associated cellular processes; Table S2: RT-qPCR results of right-sided colon cancer patients; Table S3: RT-qPCR results of left-sided colon cancer patients; Table S4: Statistical summary of gene enrichment analysis based on RT-qPCR results from right-sided colon cancer patients; Table S5: Statistical summary of gene enrichment analysis based on RT-qPCR results from left-sided colon cancer patients; Table S6: Metabolites associated with significantly differentially expressed genes identified by RT-qPCR analysis in right-sided colon cancer; Table S7: Metabolites associated with significantly differentially expressed genes identified by RT-qPCR analysis in left-sided colon cancer.

## Abbreviations

The following abbreviations are used in this manuscript:

*ANGPT2*	Angiopoietin-2
APC	Adenomatous polyposis coli
*AURKA*	Aurora kinase A
BMI	Body mass index
CIN	Chromosomal instability
CPT	Carnitine palmitoyltransferase
DSP	Desmoplakin
*EGFR*	Epidermal growth factor receptor
*ERCC3*	Excision repair cross-complementation group 3
*FDR*	False discovery rate
*FLT-1*	Fms-related receptor tyrosine kinase 1 (VEGFR1)
G6P	Glucose-6-phosphate
GPX4	Glutathione peroxidase 4
KEGG	Kyoto Encyclopedia of Genes and Genomes
MCM2	Minichromosome maintenance complex component 2
MMR	Mismatch repair
MSI	Microsatellite instability
