# Supplementary material for: Gene Expression-Based Inference of Metabolic Signatures Reveals Distinct Molecular Profiles in Right- and Left-Sided Colon Cancer
Source: Metabolites. 2025 Nov 27;15(12):768. doi: 10.3390/metabo15120768 (PMC12734406; doi:10.3390/metabo15120768)
Supplement: Supplementary file 1 [file metabolites-15-00768-s001.zip › metabolites-3965300-supplementary.pdf]

Gene Expression and Metabolite Profiling Reveal Distinct Molecular Signatures in Right- and Left-Sided Colon Cancer

Supplementary Table S1: List of primers grouped according to their associated cellular processes.

Cellular Process	Related Genes
Apoptosis	*APAF1, BIRC3, XIAP, FASLG, CASP2, CASP7, CASP9, CFLAR, NOL3, BCL2L11, TP53*
Angiogenesis	*ANGPT1, ANGPT2, FGF2, FLT1, KDR, SERPINF1, PGF, TEK, VEGFC, ADM, ARNT, CA9, EPO*
Cell cycle and proliferation	*CCND2, CCND3, CDC20, E2F4, STMN1, MCM2, MKI67, SKP2, AURKA, WEE1, BMI1, CDK4, TBX2*
DNA damage response and repair mechanisms	*DDB2, DDIT3, ERCC3, ERCC5, LIG4, POLB, GADD45G, PPP1R15A, TP53*
Signal transduction pathways	*MAPK14, ETS2, MAP2K1, MAP2K3, IGFBP3, IGFBP5, IGFBP7, SERPINB2*
Cell adhesion/EMT	*CDH2, DSP, FOXC2, KRT14, SNAI1, SNAI2, SNAI3, SOX10, GSC, OCLN*
Energy metabolism	*ACLY, ATP5A1, HMOX1, LDHA, SLC2A1, ACSL4, CPT2, G6PD, GPD2, LPL, PFKL, SOD1*
Telomere function and structure	*DKC1, TEP1, UQCRFS1, COX5A, PINX1, TERF1, TERF2IP, TINF2, TNKS, TNKS2*
Stress response	*TRAP1, CDC37, HSP90AB1, HSP90AB4P, HSPB1*

Supplementary Table S2: RT-qPCR results of right-sided colon cancer patients. The results were analyzed according to the ΔΔCt method. GAPDH and ACTINB were used as reference genes. Data are presented as mean ± standard deviation (n=6). Statistical analysis was performed using two-way ANOVA followed by multiple comparisons test, considering the control group as 1. $p < 0.05$ was considered statistically significant ($p < 0.05$, ; $p < 0.01$, *; $p < 0.001$, ***). Analyses were conducted using GraphPad Prism software version 8.0.2 (GraphPad Software, San Diego, CA, USA).

Gene	Expression Change	Gene	Expression Change	Gene	Expression Change
ANGPT1	-1.78 ± 1.64	*MAPK14*	1.52 ± 1.64	*ATP5A1*	0.87 ± 1.16
ANGPT2	-0.05 ± 2.61	*IGFBP3*	0.95 ± 1.99	*COX5A*	-0.66 ± 3.44
FGF2	-6.0 ± 5.55	*ETS2*	0.24 ± 1.6	*EPO*	1.01 ± 2.86
FLT1	-13.82 ± 37.66*	*IGFBP5*	6.3 ± 23.84	*ACSL4*	-1.21 ± 1.64
KDR	6.84 ± 17.14	*IGFBP7*	-0.7 ± 1.59	*HMOX1*	0.2 ± 1.86
SERPINF1	0.66 ± 3.68	*SERPINB2*	3.61 ± 4.18	*LDHA*	0.84 ± 1.78

PGF	-4.35 ± 5.58	*MAP2K1*	1.46 ± 2.16	*SLC2A1*	1.43 ± 1.99
CCL2	-1.49 ± 1.69	*MAP2K3*	0.73 ± 3.35	*CPT2*	1.54 ± 2.53
TEK	2.94 ± 6.46	*DDB2*	3.32 ± 2.03	*DKC1*	1.43 ± 1.93
VEGFC	2.38 ± 3.14	*DDIT3*	0.25 ± 1.83	*G6PD*	0.33 ± 1.82
APAF1	-1.41 ± 1.53	*ERCC3*	4.33 ± 5.65	*PFKL*	-2.23 ± 5.17
BIRC3	2.51 ± 1.41	*ERCC5*	-6.89 ± 19.96*	*TP53*	-1.51 ± 4.36
XIAP	1.13 ± 1.3	*LIG4*	-6.46 ± 11.38*	*UQCRFS1*	-0.58 ± 1.64
FASLG	2.3 ± 3.95	*POLB*	5.1 ± 4.66*	*TNKS2*	-0.65 ± 1.79
CASP2	-10.46 ± 13.2**	*SOD1*	2.11 ± 2.3	*GPD2*	0.79 ± 2.02
CASP7	0.64 ± 1.69	*TBX2*	1.83 ± 5.8	*PINX1*	3.61 ± 8.08
CASP9	-3.38 ± 4.47	*GADD45G*	-0.17 ± 2.67	*TRAP1*	-0.91 ± 2.74
CFLAR	-2.19 ± 3.31	*PPP1R15A*	2.68 ± 1.65	*CDC37*	3.45 ± 4.62
NOL3	-1.72 ± 3.30	*CDH2*	0.05 ± 3.23	*TINF2*	1.21 ± 1.31
BCL2L11	-3.28 ± 3.93	*DSP*	1.74 ± 3.57	*HSP90AB1*	3.48 ± 2.05
CCND2	-2.05 ± 6.3	*FOXC2*	-1.36 ± 3.69	*TERF1*	0.96 ± 2.07
CCND3	-1.19± 4.68	*KRT14*	-4.07 ± 7.65	*GUSB*	0.2 ± 2.95
CDC20	2.38± 3.11	*SNAI2*	-0.86 ± 4.41	*LPL*	3.48 ± 5.57
E2F4	-0.94 ± 1.54	*SNAI1*	-0.31 ± 3.0	*TEP1*	1.88 ± 2.34
STMN1	0.71 ± 2.93	*SOX10*	1.13 ± 3.0	*CDK4*	1.31 ± 2.86
MCM2	5.07 ± 2.43*	*GSC*	2.03 ± 2.78	*TERF2IP*	1.13 ± 1.16
MKI67	0.53 ± 2.75	*SNAI3*	-4.6 ± 4.84	*HSP90AB4P*	1.84 ± 0.65
SKP2	0.71 ± 1.73	*OCLN*	-3.52 ± 10.44	*TNKS*	0.72 ± 2.52
AURKA	-0.03 ± 3.46	*ACLY*	-0.16 ± 2.2	*HSPB1*	0.26 ± 2.45
WEE1	1.46 ± 1.81	*ADM*	1.01 ± 2.05	*CA9*	-2.13 ± 0.33
BMI1	-0.21 ± 1.51	*ARNT*	-2.17 ± 3.86		

Supplementary Table S3: RT-qPCR results of left-sided colon cancer patients. The results were analyzed according to the ΔΔCt method. GAPDH and ACTINB were used as reference genes. Data are presented as mean ± standard deviation (n=6). Statistical analysis was performed using two-way ANOVA followed by multiple comparisons test, considering the control group as 1. $p < 0.05$ was considered statistically significant ($p < 0.05$, ; $p < 0.01$, *; $p < 0.001$, ***). Analyses were conducted using GraphPad Prism software version 8.0.2 (GraphPad Software, San Diego, CA, USA).

Gene	Expression Change	Gene	Expression Change	Gene	Expression Change
ANGPT1	-0.68 ± 8.24	*MAPK14*	5.72 ± 11.07	*ATP5A1*	1.55 ± 3.61
ANGPT2	-3.34 ± 19,68	*IGFBP3*	5.91 ± 9.89	*COX5A*	2.51 ± 6.38
FGF2	-4.39 ± 9.15	*ETS2*	1.83 ± 6.92	*EPO*	3.47 ± 15.2
FLT1	-1.18 ± 12.85	*IGFBP5*	6.33 ± 7.13	*ACSL4*	-0.49 ± 4.36
KDR	-1.96 ± 7.8	*IGFBP7*	0.16 ±2.8	*HMOX1*	0.9 ± 3.87
SERPINF1	1.13 ± 10.97	*SERPINB2*	4.19 ± 9.57	*LDHA*	2.68 ± 4.53

PGF	-8.14 ± 3.97**	*MAP2K1*	3.63 ± 4.08	*SLC2A1*	2.99 ± 8.47
CCL2	-2.11 ± 7.71	*MAP2K3*	1.29 ± 2.79	*CPT2*	6.56 ± 10.74
TEK	4.60 ± 7.58	*DDB2*	6.27 ± 6.05	*DKC1*	2.98 ± 6.09
VEGFC	4.16 ± 9.28	*DDIT3*	1.39 ± 3.84	*G6PD*	2.39 ± 3.57
APAF1	0.13 ± 4.42	*ERCC3*	9.7 ± 16.41	*PFKL*	2.16 ± 5.13
BIRC3	5.43 ± 7.72	*ERCC5*	2.44 ± 5.4	*TP53*	2.71 ± 5.16
XIAP	1.68 ± 6.18	*LIG4*	-3.46 ± 5.66	*UQCRFS1*	1.31 ± 4.08
FASLG	8.15 ± 9.2	*POLB*	9.91 ± 11.77	*TNKS2*	1.55 ± 4.63
CASP2	-4.77 ± 6.07	*SOD1*	5.1 ± 7.02	*GPD2*	3.96 ± 7.45
CASP7	4.38 ± 9.37	*TBX2*	4.99 ± 6.37	*PINX1*	3.68 ± 5.4
CASP9	-1.72 ± 4.18	*GADD45G*	2.9 ± 4.89	*TRAP1*	2.0 ± 6.22
CFLAR	0.19 ± 3.38	*PPP1R15A*	8.1 ± 11.6	*CDC37*	4.28 ± 17.26
NOL3	2.31 ± 5.29	*CDH2*	5.95 ± 10.42	*TINF2*	4.1 ± 6.4
BCL2L11	0.17 ± 3.03	*DSP*	10.76 ± 9.02	*HSP90AB1*	4.24 ± 4.78
CCND2	-1.29 ± 7.85	*FOXC2*	-0.71 ± 4.92	*TERF1*	4.85 ± 8.21
CCND3	-1.05 ± 3.15	*KRT14*	-2.97 ± 6.41	*GUSB*	2.83 ± 5.07
CDC20	3.84 ± 8.1	*SNAI2*	-0.48 ± 10.29	*LPL*	9.63 ± 14.37
E2F4	-0.02 ± 2.71	*SNAI1*	2.98 ± 13.65	*TEP1*	5.98 ± 10.52
STMN1	4.33 ± 8.24	*SOX10*	4.27 ± 9.51	*CDK4*	4.99 ± 10.03
MCM2	8.08 ± 7.31*	*GSC*	2.73 ± 3.85	*TERF2IP*	4.28 ± 5.96
MKI67	4.65 ± 9.56	*SNAI3*	-1.92 ± 6.14	*HSP90AB4P*	4.09 ± 4.83
SKP2	4.77 ± 8.76	*OCLN*	1.71 ± 3.54	*TNKS*	3.67 ± 9.43
AURKA	0.21 ± 7.98	*ACLY*	0.01 ± 4.39	*HSPB1*	5.15 ± 4.46
WEE1	2.48 ± 8.97	*ADM*	2.82 ± 7.16	*CA9*	-4.86 ± 4.41
BMI1	1.06 ± 4.18	*ARNT*	0.38 ± 3.58		

Supplementary Table S4: Statistical summary of gene enrichment analysis based on RT-qPCR results from right-sided colon cancer patients. KEGG pathway enrichment was performed using the ShinyGO 0.85 platform. Pathways were ranked according to fold enrichment, and statistical significance was determined based on FDR-adjusted p-values (accessed May 25, 2025).

**Enrichment FDR**	**nGenes**	**Pathway Genes**	**Fold Enrichment**	**Pathways**
1.5E-07	6	75	43.3	P53 signaling pathway
3.5E-06	5	75	36.1	Platinum drug resistance
1.5E-10	9	137	35.5	Apoptosis
8.7E-06	5	93	29.1	Small cell lung cancer
1.5E-04	4	87	24.9	Colorectal cancer
1.7E-05	5	109	24.8	Cell Cycle

3.0E-06	6	139	23.3	HIF-1 signaling pathway
8.4E-15	15	362	22.4	TNF signalling pathway
5.5E-06	6	159	20.4	Epstein-Barr virus infection
1.5E-06	7	202	18.7	Measles
1.5E-06	7	202	18.7	PI3K-Akt signaling pathway
2.6E-07	8	236	18.3	Cellular senescence
1.8E-06	7	211	17.9	Hepatitis B
1.0E-05	6	181	17.9	Transcriptional misregulation in cancer
9.1E-05	5	158	17.1	MAPK signaling pathway
9.9E-05	5	163	16.6	Rap1 signaling pathway
1.2E-07	9	300	16.2	Lipid and atherosclerosis
1.2E-04	5	171	15.8	Focal Adhesion
2.4E-05	6	215	15.1	Ras signaling pathway
2.6E-11	14	529	14.3	Pathways in cancer

Supplementary Table S5: Statistical summary of gene enrichment analysis based on RT-qPCR results from left-sided colon cancer patients. KEGG pathway enrichment was performed using the ShinyGO 0.85 platform. Pathways were ranked according to fold enrichment, and statistical significance was determined based on FDR-adjusted p-values (accessed May 25, 2025).

Enrichment FDR	nGenes	Pathway Genes	Fold Enrichment	Pathways (click for details)
2.3E-09	8	75	37	P53 signaling pathway
7.9E-06	5	59	29.4	Endometrial cancer
7.9E-06	5	59	29.4	VEGF signaling pathway
9.0E-07	6	71	29.3	Melanoma

9.1E-07	6	72	28.9	Non-small cell lung cancer
9.3E-07	6	73	28.5	Pancreatic cancer
1.2E-06	6	77	27	Small cell lung cancer
2.0E-07	7	93	26.1	HIF-1 signaling pathway
2.8E-08	8	109	25.5	Cell cycle
8.8E-09	9	137	22.8	Apoptosis
2.6E-08	9	158	19.8	MAPK signaling pathway
3.2E-07	8	157	17.7	PI3K-Akt signaling pathway
4.4E-14	17	362	16.3	Cellular senescence
9.6E-12	14	300	16.2	Epstein-Barr virus infection
1.7E-07	9	202	15.5	Rap1 signaling pathway
2.0E-07	9	211	14.8	Ras signaling pathway
1.5E-06	8	202	13.7	Kaposi sarcoma-associated herpesvirus infection
4.4E-07	9	236	13.2	Human T-cell leukemia virus 1 infection
2.9E-06	8	223	12.5	Focal adhesion
7.0E-13	18	529	11.8	Pathways in cancer

Supplementary Table S6: Metabolites associated with significantly differentially expressed genes identified by RT-qPCR analysis in right-sided colon cancer patients were determined using the EnrichR Metabolomics Workbench Metabolite 2022 database (accessed May 25, 2025).

Index	Name	p-value	Adjusted p-value	Odds Ratio	Comibnes score
1	AMP	0.008365	0.03988	15.65	74.88
2	ATP	0.008939	0.03988	7.56	35.67
3	Palmitic Acid	0.01496	0.03988	79.17	332.73
4	Fructose 1,6-Bisphosphate	0.01496	0.03988	79.17	332.73
5	Triacylglycerol	0.01496	0.03988	79.17	332.73
6	Glycerol	0.01496	0.03988	79.17	332.73

7	CDP	0.02969	0.04926	36.53	128.46
8	UTP	0.02969	0.04926	36.53	128.46
9	ITP	0.02969	0.04926	36.53	128.46
10	Fructose 6-Phosphate	0.03178	0.04926	33.92	116.98
11	IDP	0.03386	0.04926	31.65	107.16
12	CTP	0.03802	0.04934	27.93	91.31
13	Palmitoyl-CoA	0.04009	0.04934	26.37	84.84
14	UDP	0.1194	0.1365	8.17	17.36
15	Coenzyme A	0.1923	0.2052	4.82	7.95
16	ADP	0.2513	0.2513	3.55	4.90

Supplementary Table S7: Metabolites associated with significantly differentially expressed genes identified by RT-qPCR analysis in left-sided colon cancer patients were determined using the EnrichR Metabolomics Workbench Metabolite 2022 database (accessed May 25, 2025).

Index	Name	p-value	Adjusted p-value	Odds Ratio	Comibnes score
1	3-Mercaptopyruvic Acid	0.02023	0.08426	59.48	232.02
2	Triacylglycerol	0.02356	0.08426	49.57	185.78
3	Dihydroxyacetone Phosphate	0.02356	0.08426	49.57	185.78
4	Fructose 1,6- Bisphosphate	0.02356	0.08426	49.57	185.78
5	Glycerol	0.02356	0.08426	49.57	185.78
6	sn-Glycero-3-phosphate	0.02688	0.08426	42.48	153.63
7	3-Methyl Pyruvic Acid	0.03019	0.08426	37.17	130.11
8	alpha-D-Glucose 6-Phosphate	0.03349	0.08426	33.04	112.22
9	beta-D-Glucose 6-Phosphate	0.03349	0.08426	33.04	112.22
10	Glucose 6-Phosphate	0.03349	0.08426	33.04	112.22
11	FAD	0.04332	0.08426	24.78	77.77
12	CDP	0.04658	0.08426	22.87	70.13
13	UTP	0.04658	0.08426	22.87	70.13
14	ITP	0.04658	0.08426	22.87	70.13
15	Fructose 6-Phosphate	0.04982	0.08426	21.23	63.69
16	NAD+	0.05141	0.08426	5.72	16.98
17	IDP	0.05305	0.08426	19.82	58.19
18	CTP	0.05949	0.08879	17.48	49.34
19	Palmitoyl-CoA	0.06269	0.08879	16.51	45.73
20	Glucuronic Acid	0.06588	0.08879	15.64	42.55
21	Pyruvic Acid	0.06906	0.08879	14.86	39.72
22	ATP	0.1470	0.1804	3.04	5.82
23	UDP	0.1823	0.2140	5.11	8.71
24	AMP	0.1961	0.2206	4.71	7.67
25	Coenzyme A	0.2868	0.2978	3.02	3.77
26	NADP+	0.2868	0.2978	3.02	3.77
27	ADP	0.3674	0.3674	2.22	2.22
